# An 'Importance' Map of Signs and Symptoms to Classify Diabetic Polyneuropathy: An Exploratory Data Analysis

**DOI:** 10.1371/journal.pone.0129763

**Published:** 2015-06-15

**Authors:** Isabel C. N. Sacco, Eneida Yuri Suda, Vincent Vigneron, Cristina Dallemole Sartor

**Affiliations:** 1 Department of Physical Therapy and Speech and Occupational Therapy, School of Medicine, University of São Paulo, São Paulo, Brazil; 2 Informatique, Biologie Intégrative et Systèmes Complexes, Université d'Evry, Evry, France; Iran University of Medical Sciences, IRAN, ISLAMIC REPUBLIC OF

## Abstract

**Aims/Hypothesis:**

Early diagnosis of diabetic polyneuropathy (DPN) is critical for a good prognosis. We aimed to identify different groups of patients, based on the various common clinical signs and symptoms of DPN, that represent a progressive worsening of the disease before the onset of plantar ulceration or amputation. We also sought to identify the most important DPN-related variables that can discriminate between groups, thus representing the most informative variables for early detection.

**Methods:**

In 193 diabetic patients, we assessed 16 DPN-related signs, symptoms, and foot characteristics, based on the literature and the International Consensus on the Diabetic Foot. We used multiple correspondence analysis and the Kohonen algorithm to group the variables into micro and macro-classes and to identify clusters of patients that represent different DPN conditions.

**Results:**

Four distinct groups were observed. One group showed no indication of DPN. The remaining groups were characterized by a progressive loss of the vibration perception, without a worsening of symptoms or tactile perception. The 2 intermediate groups presented different aspects of DPN: one showed mostly DPN symptoms and the other showed the incipient vibration impairment, callus and crack formation, and foot arch alteration. The fourth group showed more severe foot and DPN conditions, including ulceration and amputation, absence of vibration and tactile perception (irrespective of how many compromised foot areas), and worse foot deformities and callus and crack formation.

**Conclusion:**

Vibration perception was more informative than tactile sensitivity in discriminating early DPN onset because its impairment was evident in more groups. Symptoms and callus and cracks did not discriminate the severity status and should be interpreted in association with other clinical variables. Reconsideration of the current screening techniques is needed to clinically determine the early onset of neuropathy using tactile perception.

## Introduction

Diabetic polyneuropathy (DPN) remains a challenge for patients and clinicians. Early diagnosis is recommended and is the key factor for a better prognosis [1], but there are few theoretical and clinical studies dedicated to this task. DPN has an insidious and non-homogeneous manifestation, making it difficult to identify its onset [2]. Most attention is given to patients with a high risk of ulcer formation, in whom the disease is usually already considered severe. Less attention is given to patients with mild disease severity but who, long before ulceration and amputation occur, are already undergoing tissue and nerve damage. Although the International Consensus on the Diabetic Foot [1] pays great attention and recommends specific tests to identify patients at risk for foot ulceration, individuals with different levels of impairment should also be targets of preventive health actions to try to avoid the devastating consequences of DPN.

The most common available tools used for clinical diagnosis of DPN are validated questionnaires for the identification of specific signs and symptoms, physical examination of the foot, and sensorial tests that assess neurological impairments [1–4]. The somatosensorial modalities frequently assessed and recommended by the International Consensus on the Diabetic Foot for DPN detection are pressure sensation under the plantar surface, tested with 10-g Semmes-Weinstein monofilaments [5], and vibratory perception, tested with a biothesiometer or 128-Hz tuning fork [6,7]. Particularly, the perception of 10-g monofilament has a high reproducibility and specificity and may be used to predict ulceration and amputation risks [8,9]. A set of common DPN symptoms, such as tingling, burning, numbness, and prickling, can be asked systematically of patients using validated questionnaires to detect impairments in small fibers [10]. A physical examination of the foot to detect dry skin with crack or callus formation may indicate associated autonomic neuropathy that also predisposes to ulceration [10,11]. Inspection for foot deformities, such as claw or hammer toes, hallux valgus, and low or high foot arches, are also important because these structural alterations predispose foot areas to excessive loads and tissue breakdown [12]. Although electroneuromyography is the gold standard for DPN diagnosis, when aiming to improve clinical assessment, information about clinical DPN-related variables is desirable to prevent the patient’s exposure to an uncomfortable and invasive procedure.

Currently available tools and questionnaires used to classify DPN severity [13–18] do not necessarily make clear what this pool of DPN-related variables mean when different associations between them are made in a clinical setting. Because the disease manifestation is heterogeneous among patients, it is difficult to classify the disease status, and different associations among those DPN-related variables may have diverse meanings. In addition, each variable may need to have a different weight in the diagnosis and classification of DPN severity. Using a simple sum of DPN-related variables commonly used in questionnaires [13,15–17] may under- or overestimate the disease status. How important is each clinical variable in the DPN diagnosis and classification? A better understanding of these associations and their importance would contribute to disease prevention by recognizing clusters of patients according to DPN status. Improving patient classification is important not only for researchers, where it is crucial to have homogeneous groups when investigating other parameters, but also for clinicians who can then choose the most appropriate care for every patient and, most importantly, to detect early impairments. An appropriate and deeper analysis can indicate the most important variables related to each cluster of patients and therefore provide a new approach to classify DPN severity.

Patients with differing levels of impairment were the focus of our study in which we aimed to determine among a composite of common signs and symptoms of DPN, different cluster groups of patients. We also sought to identify the most important DPN-related variables that can discriminate between clusters, thus representing the most informative variables.

## Proposed Qualitative Data Analysis

### Participants

Patients were recruited from 3 settings: (1) a diabetes mellitus ambulatory medical care unit located in a regional hospital, (2) the National Association of Diabetes, and (3) a primary care center of the School of Medicine of the University of Sao Paulo. A total of 193 patients were included in this study (Table 1, females 53.9%). All procedures were approved by the School of Medicine Ethics Committee, University of Sao Paulo (protocol 0305/08; CAAE—0131.0.015.000–10), and the participants gave written informed consent.

**Table 1:** Sample demographic and anthropometric data.

Variable	Mean ± SD
**Age (years)**	57.40±6.21
**Body mass index (kg/m^2^)**	28.72±4.73
**Time of disease (years)**	10.50±8.92

The eligibility criteria were patients who were 45–65 years of age and had diabetes mellitus type 1 or 2, body mass index ranging 18.5–29.9 kg/m^2^ (normal and overweight classifications), and no partial or total foot amputation. Patients were not selected if they had other neurological or orthopedic impairments not caused by DPN, major vascular complications, or severe nephropathy.

### Clinical assessment

Symptoms of DPN were assessed based on the standardized questionnaire of the Michigan Neuropathy Screening Instrument [19]; of the 15 questions, we included 10 that were considered the most specific for DPN identification. The foot physical examination was performed visually to identify the presence of common signs and structural alterations that develop with DPN, such as callus, cracks, hallux valgus, hammer toes, claw toes, cavus or flat foot. Any history of foot ulceration or amputation was also questioned.

Tactile perception was assessed with Semmes-Weinstein monofilament of 5.07/10 g applied to a non-callused site under 4 areas of both feet (total of 8 foot areas): plantar surface of the distal phalanx of the hallux and the first, third, and fifth metatarsal heads. The examiner applied the stimulus arrhythmicially and in a random order, the patient was asked to report the areas where the stimulus was felt [5,6].

Vibration perception was assessed by a timed method, according to Perkins et al [6], with a 128-Hz tuning fork applied bilaterally to the bony prominence of the interphalanx joint of the hallux. The patient reported when the perception of the vibration was not felt anymore. The examiner then noted the time (in seconds) that vibration sensation diminished beyond the examiner’s perception in the hand holding the tuning fork. Under 10 seconds was considered normal, and longer than 10 seconds was considered diminished. Perception was considered absent if the patient did not report feeling the vibration.

All clinical tests and exams (Table 2) were performed by the same trained assessor.

**Table 2:** Symptoms and signs assessment, qualitative DPN-related variables for the study and variables modalities.

Symptoms	Evaluation result	Variable	Variables modalities
***Distal leg symptoms***			
Are you legs and/or feet numb?	Yes	Numbness	Numbness 1
	No		Numbness 2
Do you ever have any burning pain in your legs and/or feet?	Yes	Burn	Burn 1
	No		Burn 2
Do you ever have any prickling feelings in your legs or feet?	Yes	Prick	Prick 1
	No		Prick 2
Do you ever have any stinging feelings in your legs or feet?	Yes	Needle	Needle 1
	No		Needle 2
Does it hurt when the bed covers touch your skin?	Yes	Sheet	Sheet 1
	No		Sheet 2
Are your symptoms worse at night?	Yes	Night	Night 1
	No		Night 2
Are your symptoms worse at rest?	Yes	Rest	Rest 1
	No		Rest 2
Are you able to sense your feet when you walk?	Yes	Walk	Walk 1
	No		Walk 2
***History of foot ulceration***			
Have you ever had an open sore on your foot?	Yes	Ulcer	Ulcer 1
	No		Ulcer 2
***History of foot amputation***			
Have you ever had an amputation?	Yes	Amput	Amput 1
	No		Amput 2
***Foot inspection***			
Hallux valgus	Absent	Hallux	Hallux 1
	Present		Hallux 2
Plantar arch	Normal	Arch	Arch 1
	Flat foot OR cavus foot		Arch 2
Claw toe OR hammer toe	Absent	DeformToes	DeformToes 1
	Present—One foot		DeformToes 2
	Present—Both feet		DeformToes 3
Skin cracks OR callus	Absent	CalusCrack	CalusCrack 1
	Present—One foot		CalusCrack 2
	Present—Both feet		CalusCrack 3
***Somatossensorial perception***			
Pressure sensation	Preserved	Pressure	Pressure 1
	Diminished in 1 or 2 areas		Pressure 2
	Diminished in 3 to 2 areas		Pressure 3
	Diminished in 6 to 8 areas		Pressure 4
Vibration sensation	Preserved		Vibration 1
	Diminished in one OR in both feet	Vibration	Vibration 2
	Absent in one foot OR diminished in one foot and absent in the other foot		Vibration 3
	Absent in both feet		Vibration 4

We transformed the patient’s evaluated characteristics into categorical variables to allow mathematical analysis (Table 2) and determined a set of modalities for each variable. For the symptoms, history of foot ulceration and foot amputation, and hallux valgus, 2 modalities were established: yes (when present) or no (when absent). Plantar arch was classified as normal or cavus/flat. Claw or hammer toes and callus or cracks were classified in 3 modalities: absent, present in 1 foot, or present in both feet.

Tactile and vibration sensitivity were divided into 4 modalities. For tactile perception, we used the following classification: preserved, diminished in 1 or 2 areas, diminished in 3 to 5 areas, diminished in 6 to 8 areas. Vibration perception was classified as preserved, diminished in 1 or both feet, absent in 1 foot or diminished in 1 foot and absent in the other foot, and absent in both feet.

All of the variables included in our analysis are described in more detail in Table 2.

### Factorial correspondence analysis and multiple correspondence analysis

The multiple correspondence analysis (MCA) is a type of factorial analysis capable to analyze observations described by nominal variables. Each categorical DPN-related variable comprises several levels, and each level is coded by a binary value (see Table 2). For instance, hallux valgus is one nominal variable with 2 levels (Table 2). The variable toe deformity will be '0 1' and '1 0' in the other case. MCA can accommodate with quantitative variables by recording them as bins. A score of -1 to +1 could be recorded by a nominal variable with 3 levels: <-0.5, between -0.5 and +0.5, and >+0.5. Algebraically MCA can be seen as a generalization of principal component analysis when the variables to be analyzed are qualitative instead of quantitative, i.e. when the matrix to be analyzed is an indicator matrix (i.e. a matrix with entries of 0 or 1), a complete disjunctive table, or a Burt table [20]. The additional binary columns created artificially for each variable induce additional dimensions, which is of uppermost interest for clustering the data.

Like any ordination method, MCA's purpose is to find the best summary in a space of reduced dimension which consists of a small number of axes that maximizes the projected inertia of the cloud of data points.

The MCA interpretation is based on proximity between points in a low dimensional space, in 2 or 3 dimensions on the total number of dimensions. Proximities are significant only between points of the same series *(ie* lines with lines, columns with columns). For instance, if there is a considerable proportion of individuals (row points) who select modality “a” of question “1” and modality “b” of question “2”, then we say that the modalities (1,a) and (2,b) attract each other [21, 22]. We expect them to be very close in the representation. Conversely, if there is a substantial proportion of individuals who choose (1,a) and reject (2,b), then these modalities repulse each other, and we will observe distant representation. The goal is to represent these kinds of observations in a global manner that takes into account all the modalities of all variables. The proximity between variable levels indicates that the groups of observations associated with these 2 levels are similar. Further, a subset of modalities will be closer to a subset of individuals that have chosen these modalities than a group of individuals that have not.

**Fig 1:**
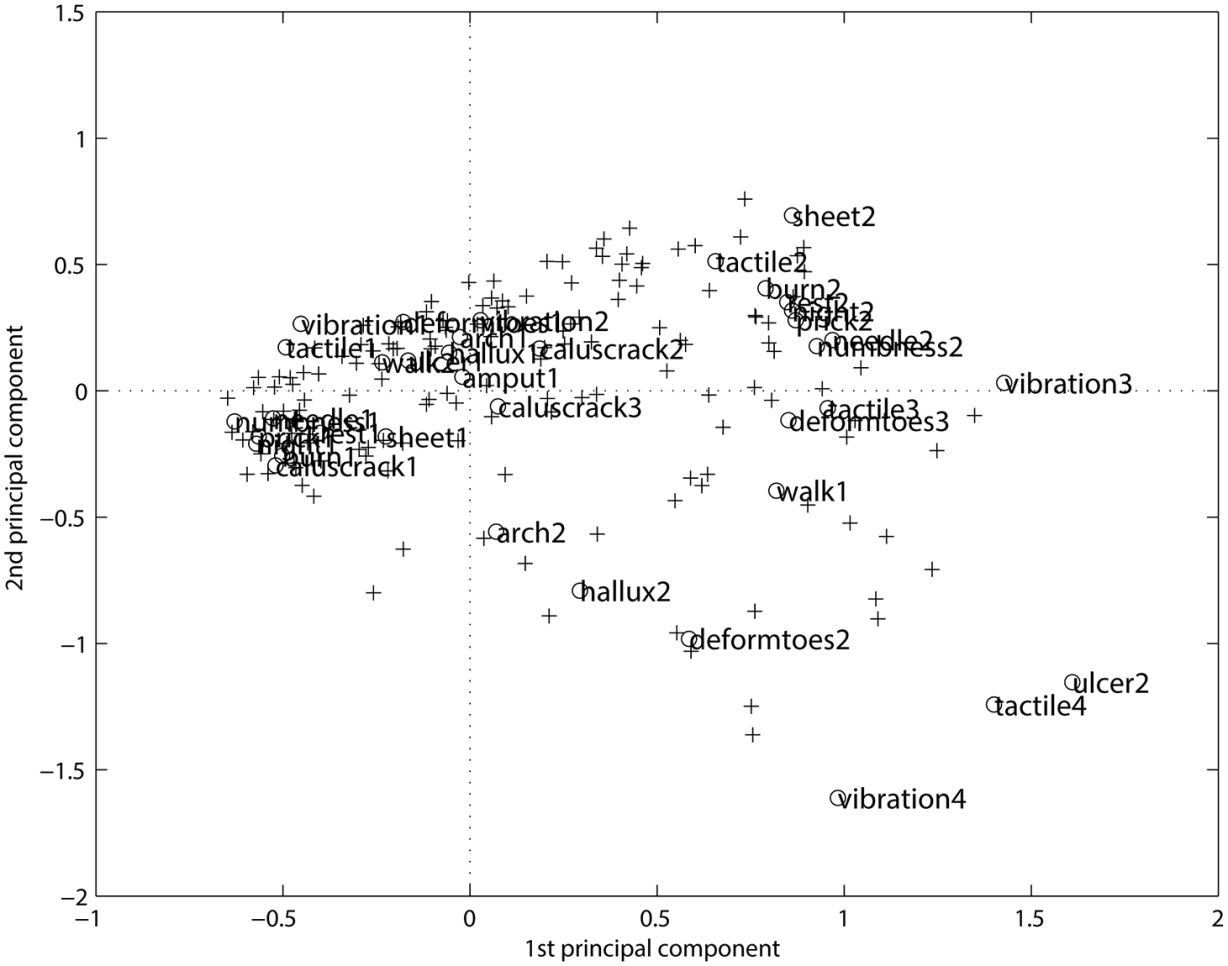
The MCA representation. The projection on the first 2 axes (29%) and 2 (17%) represents only 46% of the explained inertia from Table 2. The ‘+’ input is for the rows (i.e. patients) and the ‘o’ input is for the columns (i.e. symptoms).

From the MCA analysis of Table 2, it is possible to represent simultaneous rows and columns, i.e. of individuals ('+') and variables ('o'), in a space of reduced dimensions, as shown in Fig 1. The Fig 1 separate the first modalities of the variables, clustered on the left negative side from the second and third modalities on the right positive side. The positive side gives the reverse pattern with respect to the negative side. As the modalities are classified according to some (irregular) scale, the progression from level 1 (the best) to the worst (level 3, or 4, or 5, depending on the modality) is clearly visible. But the clusters are not clear, as well as the association among patients and symptom categories.

MCA is a linear projection method and provides several 2-dimensional maps. It is therefore necessary to consider several cards at once if the categories are more or less represented, and it is not always easy to deduce relevant conclusions on the proximity between the categories. In addition, related modalities should be projected onto neighboring points, but frequently distortion due to the linear projection affects the visualization. Therefore, the representation of the information provided by this classification is often unsatisfactory.

A visual tool that would both organize the neighboring classes together and explain the layout of these classes in the input space could greatly improve the interpretation of classifications. A classification method is often coupled with a factor analysis, but the resulting solution space is artificially inflated, underestimating seriously the percentage of inertia explained by the first dimension, which makes projections on the factorial plans and their interpretation risky.

### From MCA to Kohonen representation

When standard linear mappings are not suitable due to the nonlinear structure of the data, one can try to use artificial neural networks because they are intrinsically nonlinear such as the Kohonen algorithm [23], which is widely used for data analysis. See also [3, 4, 5, 23, 24], where the method is fully described and explained. The Kohonen algorithm, also called Self-Organizing Maps (SOM) [24], is used to group and rearrange the observations like any classification method [25] but offers the originality *(i)* to visualize the neighborhood structure between classes *(ii)* to duplicate the topology of the input space. The main tool is a network made up by *n* × *n units* arranged following a 2-dimensional grid for which each unit is *u* characterized by a “code-vector” ***c*** *_u* with the same dimension as the input space. Consider an array of *N* observations where each individual is described by *q* qualitative variables. The estimation of the code vectors, an unsupervised form of statistical learning, is not addressed in this paper. It is supposed to be successfully performed and the “natural” structure inherent in the input date captured. The Kohonen network maps the input vectors onto a discrete map with 2 dimensions in which patterns close to one another are topologically ordered. At the end, each individual is assigned by a nearest-neighbor algorithm to the class *u* if the code vector **c**_u the closest among all the code vectors in the sense of the Euclidean distance.

The main property of the Kohonen algorithm is the topology conservation of the input space: the first and raw result we get after learning is that close observations are associated with the same class or close classes as defined in the neighborhood in a Kohonen network. The resulting classification can be considered as a good starting point for further development.

The data matrix is the *Burt* table [25] obtained by the inner product of the previous binarized data table. The Burt table has one column and one row for each level (category) of each categorical variable. After the neural model training, the units of the network are the representative of the rows of the Burt matrix and topologically organized as a classification of all the modalities, respecting the topology-preserving constraint on related modalities belonging to the same class or to neighboring classes, which is easy to visualize and can be further clustered into macro-classes.

**Fig 2:**
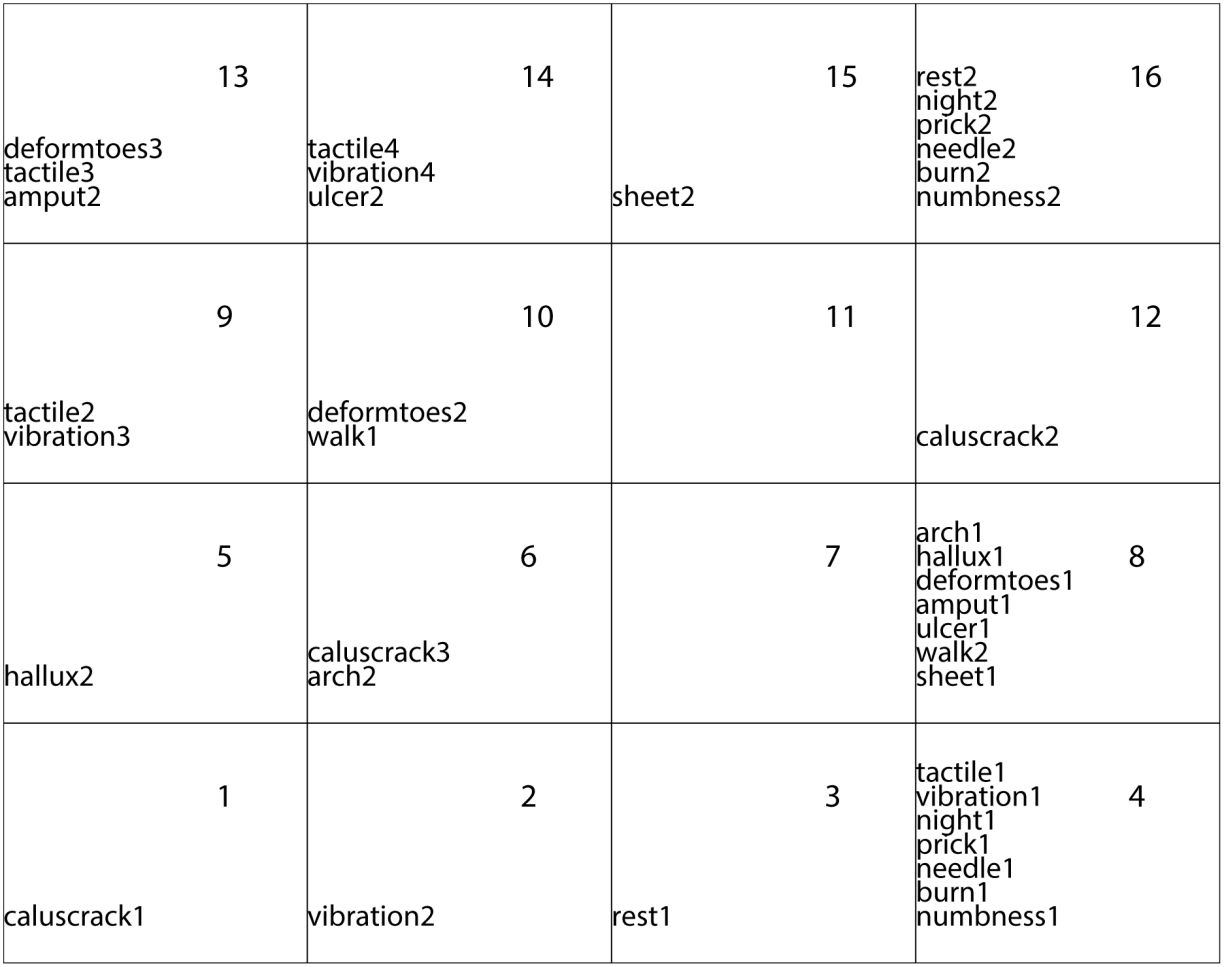
Repartition of the 39 modalities on the Kohonen map with 500 iterations.

Fig 2 represents the result of the Kohonen algorithm applied to the DPN data. It is easy to see that the “good” modalities (level 1) are *mainly* grouped together in the bottom right-hand corner followed by the “intermediate” modalities (level 2), in the top left-hand corner. The “bad” modalities (level 3) are displayed in the right-hand corner. There are one empty class and one with only one “good” variable that separate roughly the best modalities from the worst ones.

When comparing the map in Fig 2 with the projection in Fig 1 provided by MCA, we guess similarities in the global display. The first axis of the MCA opposes the best modalities (on the bottom) to the worst ones (on the top) but with a loss of information (only 46% of explained inertia). It is necessary to consider the first 9 axes to keep more of the three-quarters of the total inertia. The transition in Fig 1 from level 1 to level 3 is evident, but the clusters are not clear. Some contradictory conclusions could be even observed when examining the further axes.

In the following, we show (1) how to perform a robust classification based on ***categorical*** information, (2) how to visualize the classes and their differences and homogeneities, and (3) how to put in evidence the most explanatory variables.

### Two classification levels

**Fig 3:**
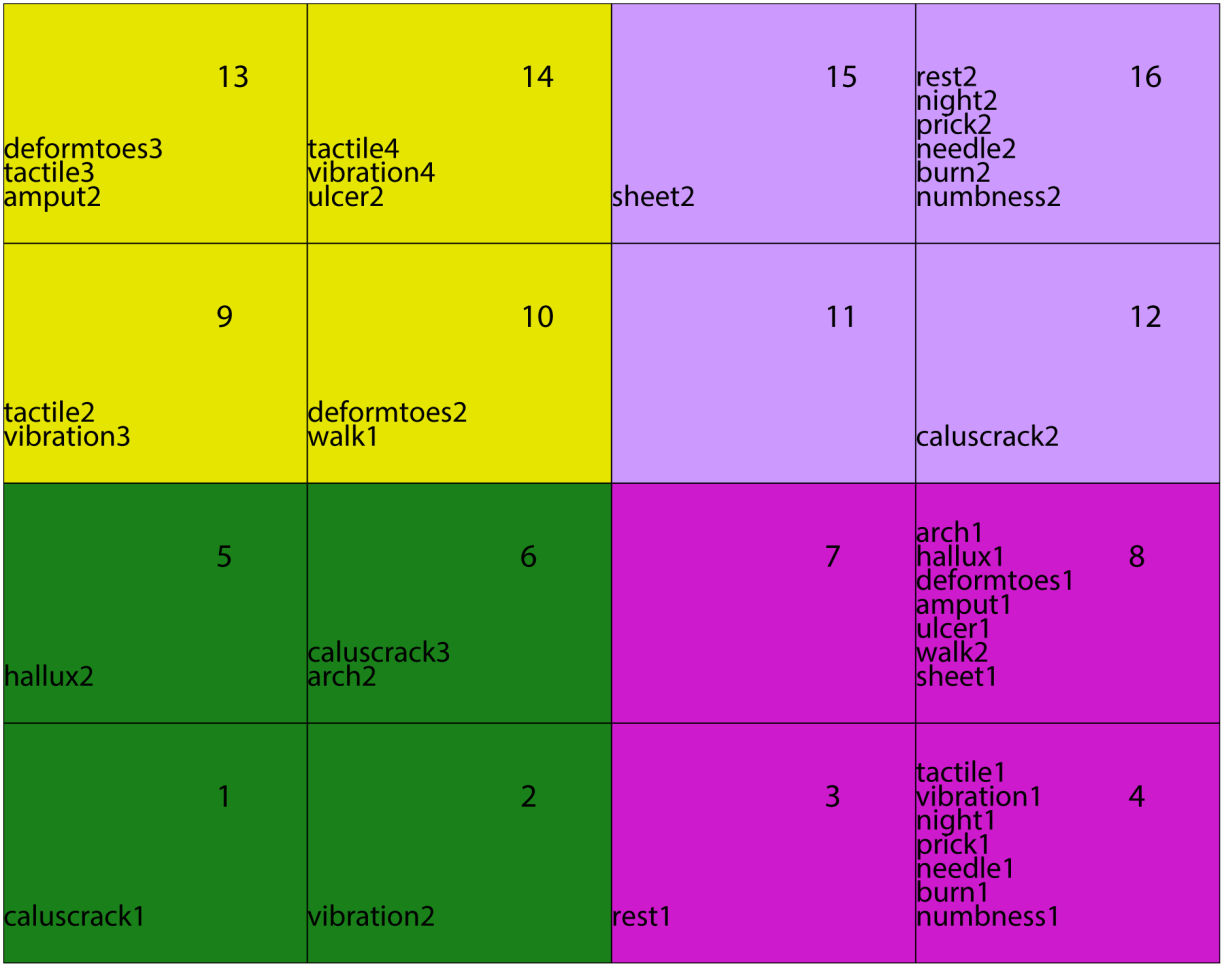
Macro-classes grouping the modalities into 4 easy-to-describe classes.

There exists no method to choose the network's size, hence a arbitrary number of units *p* is chosen. We can only guess that the relevant number of classes will be smaller than *p*. In addition it is difficult to interpret a too large number of classes. Therefore we suggest to lower the number of classes (of units) with the aid of a hierarchical classification of the *p* code vectors which is relevant according to the grid organization of the units. We design two embedded classifications using the Ward distance [26]: one composed of micro-classes (Kohonen classes) and the other of macro-classes that group together some of the micro-classes. To visualize this 2-level classification, we assigned each macro-class a color or gray level (Fig 3). This double classification offers the possibility to visualize at a macro level the main general features and at a micro level to zoom on the attributes of more local embedded phenomena, especially the path to go from one class to another. Moreover, macro-classes produce connected areas in the grid organization, which confirms the topology-preserving properties of the map. A representation of the micro-classes grouped together to constitute 4 macro-classes is given Fig 3. Eventually, some classes may be empty.

### Analysis of the classes: discrimination and homogeneity

After the classes have been formed, any standard criterion can be computed to measure the inter- and intraclass variances, *i*.*e*. to measure respectively the *intra*-cluster homogeneity, *inter*-cluster separability [24]. To improve visual inspection of the grid topology, we propose some graphical tool for visualizing the distance between the clusters.

**Fig 4:**
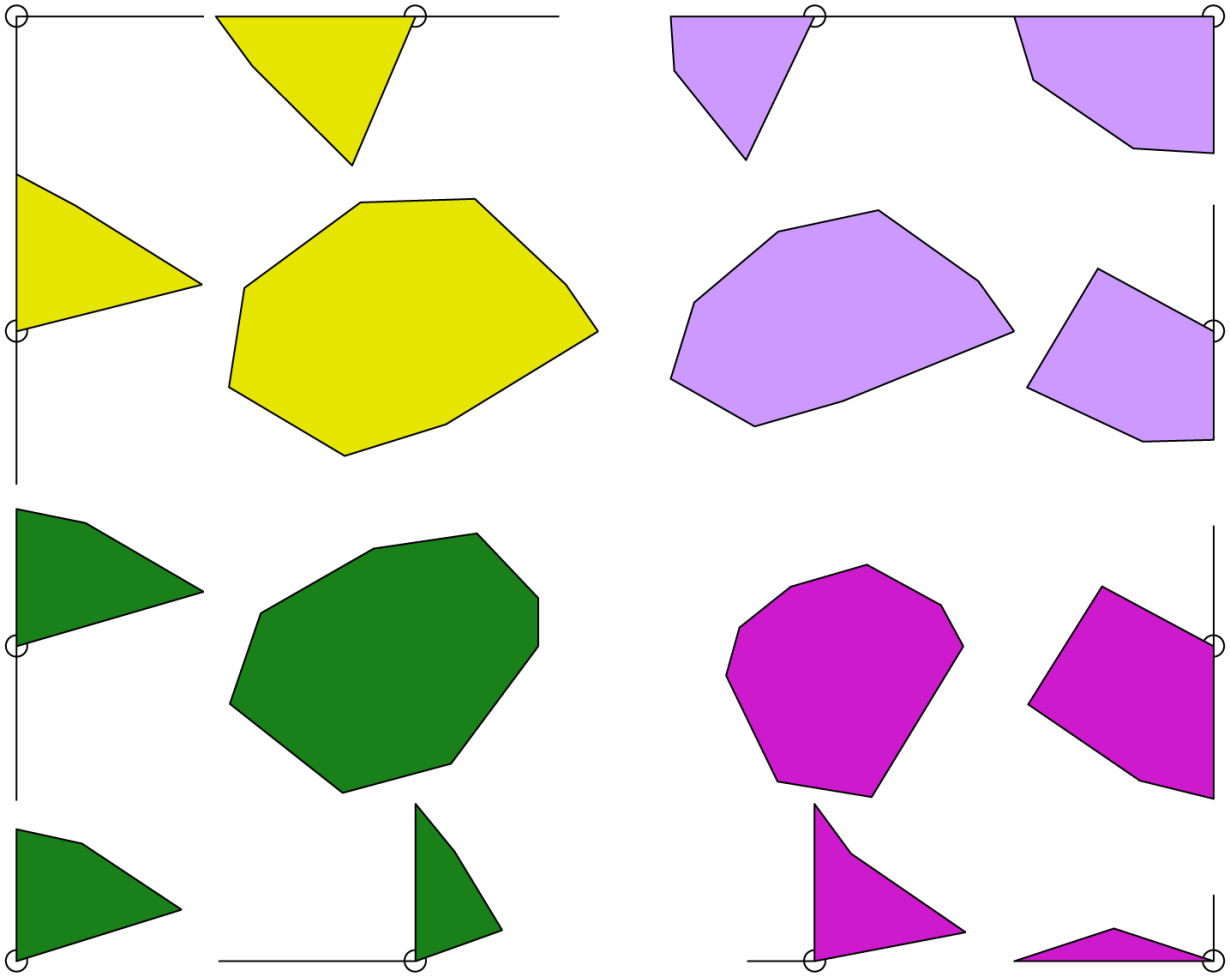
Representation of the distances in the Kohonen map. The gaps coincide with the fontiers of the macro-classes (colored).

The standard representation of the (square) grid is a regular organization of the units as in Fig 3, but the code vectors draw a *q*-dimensional surface with irregular distances between the classes. This deviation between the representation and the mathematical fact can source confusion in the interpretation. The weakness of the representation lie in the missing visualization of the distances between classes. This visualization would help to prevent misleading interpretation and would explain the discrimination between the classes. For this we modelize each micro-class by an octagon. By convention, the larger it is, the closer the unit is to its neighbors. Therefore, clusters are regions in which neighbor octagons tend to be large and frontiers are regions which largely do not intersect (Fig 4). In general, we can observe that the boundaries of macro-classes coincide with greater distances between classes, and this observation confirms the relevance of the second level of classification. On the oppposite, if a boundary is produced between two small distance classes, this means that the second classification level merges the two sub-groups into a single group and that the path from one to another is continuous. It points out that we might probably consider a hierarchical classification with less classes.

**Fig 5:**
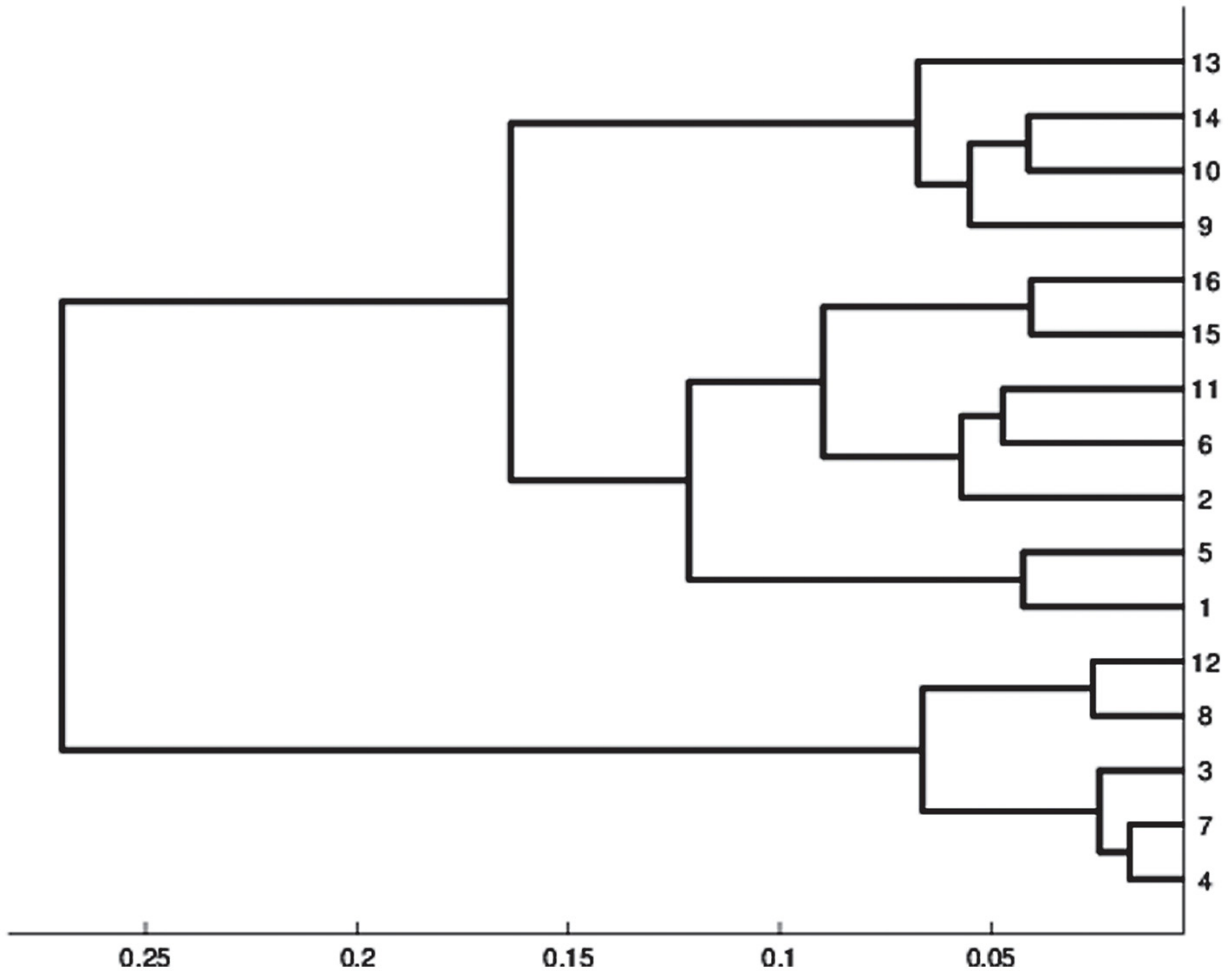
Hierarchical classification. The y axis indicates the macro-classes that are represented in Figs 2 and 3.

The decision of grouping the sample in a determined set of macro-classes was established according to the author’s purpose of detecting early stages of DPN manifestation and the main characteristics of this progression rather than just identifying the absence or presence of DPN. Therefore, an extensive data observation was performed to find the best grouping criteria. Using the Kohonen map with the represented distances between macro-classes (Fig 4) and the cluster graph (Fig 5), we can observe that 4 macro-classes or large groups could be identified and could represent different DPN stages or conditions.

The first macro-class (group) that was identified presented the common characteristics of absence of DPN symptoms, absence of foot deformities, absence of callus and cracks, and preserved tactile and vibration perception, therefore representing a healthy group without any sign of DPN (Fig 4, green).

In the second macro-class (group), all the symptoms of DPN, when present, were grouped together, except for the absence of feeling the feet touching the ground while walking. In addition, callus and cracks were present and vibration perception was absent for 1 foot only, indicating a mild neuropathy (Fig 4, magenta).

The third macro-class (group) was characterized by a further worsening of vibration perception and an absence of callus and cracks or their presence in both feet, suggesting those particular variables are not representative of DPN aggravation (Fig 4, violet).

Finally, the fourth macro-class (group) clearly represents the worst DPN condition, being characterized by absence of vibration perception in both feet, foot deformities, history of foot amputation and ulceration, and interestingly, absence of tactile perception regardless of being in a small portion of one foot or on all plantar surfaces of both feet (Fig 4, yellow).

Table 3 shows the proportion of patients that were allocated in each of the variable modalities that composed the MCA model.

**Table 3:** Frequency distribution of patients that were allocated in each of the DPN related variables modalities.

Symptoms	Evaluation result	% (N)
***Distal leg symptoms***		
Are you legs and/or feet numb?	Yes	40.4 (78)
	No	59.6 (115)
Do you ever have any burning pain in your legs and/or feet?	Yes	38.9 (75)
	No	61.1 (118)
Do you ever have any prickling feelings in your legs or feet?	Yes	39.4 (76)
	No	60.6 (117)
Do you ever have any stinging feelings in your legs or feet?	Yes	35.2 (68)
	No	64.8 (125)
Does it hurt when the bed covers touch your skin?	Yes	20.7 (40)
	No	79.3 (153)
Are your symptoms worse at night?	Yes	39.9 (77)
	No	60.1 (116)
Are your symptoms worse at rest?	Yes	32.1 (62)
	No	67.9 (131)
Are you able to sense your feet when you walk?	Yes	77.7 (150)
	No	22.3 (43)
***History of foot ulceration***		
Have you ever had an open sore on your foot?	Yes	9.3 (18)
	No	90.7 (175)
***History of foot amputation***		
Have you ever had an amputation?	Yes	1.6 (3)
	No	98.4 (190)
***Foot inspection***		
Hallux valgus	Absent	83.9 (162)
	Present	16.1 (31)
Plantar arch	Normal	72.5 (140)
	Flat foot OR cavus foot	27.5 (53)
Claw toe OR hammer toe	Absent	77.2 (149)
	Present—One foot	21.2 (41)
	Present—Both feet	1.6 (3)
Skin cracks OR callus	Absent	21.8 (42)
	Present—One foot	49.2 (95)
	Present—Both feet	29.0 (56)
***Somatossensorial perception***		
Pressure sensation	Preserved	67.9 (131)
	Diminished in 1 or 2 areas	10.9 (21)
	Diminished in 3 to 5 areas	7.8 (15)
	Diminished in 6 to 8 areas	13.5 (26)
Vibration sensation	Preserved	52.3 (101)
	Diminished in one OR in both feet	27.5 (53)
	Absent in one foot OR diminished in one foot and absent in the other foot	6.7 (13)
	Absent in both feet	13.5 (26)

## Discussion

Our study provides a satisfactory overview of the distribution of the most common signs and symptoms that are assessed following the International Consensus on the Diabetic Foot. In addition, it was possible to determine that most of the symptoms do not discriminate between DPN severity statuses, except for the absence of sensation in the feet while walking. Tactile sensitivity could not discriminate the early stages, raising some questions about the importance of 10-g monofilament screening for preventive actions. In contrast, vibration perception was one of the most important variables that discriminated different disease statuses and macro-classes. Foot inspection is an important aspect to be addressed but could also not discriminate the severity of DPN, possibly because calluses, cracks, and toes deformities can be present before disease manifestation or even without a diabetes diagnosis.

The 4 groups we observed had very different main characteristics. It was possible to identify a group that was completely healthy, without any indication of DPN manifestation or foot alterations. The remaining groups were characterized by a progressive worsening of vibration perception, but not a worsening of the DPN-related symptoms and tactile perception. The two intermediate macro-classes were characterized by different aspects of DPN, one mostly by the symptoms of DPN and the other mainly by the insipient vibration impairment. The fourth macro-class was characterized by the DPN-related variables that indicate a more severe feet condition, such as history of ulceration and amputation, absence of vibration and tactile perception, and worse feet deformities.

Considering the variables that represent the absence of DPN (preserved tingling, burning, numbness, prickling, and tactile and vibration sensitivity), they were all grouped together in the same macro-class. Thus, the MCA together with the Kohonen methods were successful in discriminating the known conditions of the diabetes status, before and after DPN diagnosis.

When we consider only the symptoms related to DPN, specifically the tingling, burning, numbness, and prickling, they were grouped together in the same macro-class. It means that those symptoms do not provide different information that could better discriminate a more severe status of DPN. We can assume that a patient who presents just 1 of those symptoms will be classified as the same status as a patient having more than 1 of those symptoms. In other words, having more than 1 symptom does not mean a worse condition because they all represent the same phenomena of nerve impairment.

Only 1 symptom was clustered in the more severe group of patients, which was the absence of sensation of the foot touching the floor while walking, indicating not only the absence of tactile sensitivity but also the absence of pressure and thermal perception and thus reflecting a more severe neural impairment. This symptom was clustered apart from all others and represents important information to identify patients with a worse condition.

It is reasonable to think that if a patient loses sensation in more than 1 plantar area, it would represent a worse condition of DPN. Our analysis showed that irrespective of the number of compromised plantar areas, patients were grouped in the same macro-class. This was an unexpected result and raises some concerns about this method. Although it has been demonstrated that not feeling the 10-g monofilament in a specific plantar area represents 7 times more risk for plantar ulceration [8], this result is not sufficient to determine how early in the disease the sensitivity is being compromised. If a loss in 1 area only is enough to group patients in the most severe neuropathy condition, this might not be a good test to perform when we are interested in early detection and preventive actions. We tested 1 more area (head of third metatarsal) than the number recommended by the Consensus [5] (around the first and fifth metatarsal heads and distal hallux plantar surface). The 10-g monofilament evaluation is a good tool to assess the loss of protective sensation related to diabetic neuropathy [5] and has high reproducibility and specificity. The increased pressure threshold assessed by this monofilament has been demonstrated to accurately predict patients who already have a plantar ulcer or its imminent formation [8]. Since this relationship has been established, the recommendation for clinicians to use 10-g monofilament to identify patients at risk for ulcer formation has spread and is part of the practical guidelines for diabetic foot prevention [5]. Sosenko et al [8] suggested that the cutoff values that we found to be effective for the detection of foot ulcer patients may not be optimal for foot ulcer prevention programs, because the sensitivity impairments that may have started long before would not have been detected by the 10-g monofilament. As our results also suggest, pressure thresholds lower than 10 g should be tested for early detection in patients with a lower degree of sensitivity losses. Of course, in those cases, attention should be paid to skin conditions, such as callus and thickening of the plantar surface, which could highly affect the test results. Still, assessment for early identification of loss sensation should not be disregarded.

Vibration perception loss is typical in DPN progression, and in our results, it was related to 3 of the 4 macro-classes. It is important to highlight that our vibration perception test was more detailed than the one recommended by the Consensus. We assessed not only for the presence or absence of the stimulus but also for its decreased perception. And indeed, it was possible to discern a relationship between the degree of vibration loss and most of the severity groups. If we consider only the absence or presence of vibration perception, it behaves like pressure sensation and would be related only to the more severe macro-class of patients. Thus, a more detailed vibration perception assessment is important in all diabetic patients and is capable of showing early stages of the disease.

Foot inspection for joint misalignments and deformities is an important part of diabetic foot assessment because they are highly related to ulcer formation [27,28]. Claw toes, hammer toes, and hallux valgus are examples of conditions that can cause prominences of metatarsal heads and phalanx joints and thus promote areas of higher tissue stress and breakdown, mainly during dynamic activities. They also should be assessed and related to the shoe type and whether it is appropriate for the shape of the patient’s feet. However, in our analysis, it was not possible to establish a DPN status classification considering only foot deformities. Because those alterations are not exclusive to DPN, they could be present even before manifestation of the disease and not necessarily pathological. We conclude that identifying those alterations without considering other variables will not help to identify DPN status. But our results also showed that if those deformities are present in the most severe patients, they are clearly associated with the severity of the DPN.

Other very important foot alterations related to the disease are callus and crack formations. Although these alterations can also expose the foot to increased tissue loading and contribute to infections and ulcer development, this DPN-related variable did not help in discriminating macro-classes of DPN severity. Because worse and preserved feet are grouped together (macro-class 3), those variables cannot discriminate the severity status.

As expected, amputation and ulcer formation were associated in the macro-class with more severe patients. The absence of sensation of the foot touching the floor during walking was also in the same macro-class as well the absence of vibration and tactile perceptions in all plantar areas, which are the most typical symptoms and signs of DPN.

This study has some limitations. Unfortunately, it was not possible to include other assessments such as ankle reflex and hemoglobin A1c levels, because those data were unavailable for most of the patients included in this study. Because our intention with this work was to improve the interpretation of the clinical assessment by extracting, from a combination of DPN-related variables, qualitatively important information that most clinicians have access to, we did not use electroneuromyography data.

Taking into account the current literature and the Consensus recommendation, we conclude that most attention is given to patients with imminent foot ulceration rather than attempting to develop and improve assessment techniques to detect early impairments. The main concern in those studies is detecting the risk of foot ulceration, when an effort should also be made to detect patients at risk of developing DPN. The main contribution of this study is questioning the traditional method of the 10-g pressure perception threshold as a screening technique for early detection when it is already too late, and the vibration perception is far more discriminant. If different monofilament sizes are used, they could probably better discriminate the disease status, as the vibration tests do. In addition to the considerable subjectivity of both methods of assessing sensitivity, they are unquestionably clinical resources that can contribute to early detection of DPN. Future studies should focus on developing assessment strategies and tools that better detect early foot changes and instilling this habit in healthcare providers.

We propose a general methodology to analyze multidimensional data when the observations are described by clinical variables. Many other techniques can be combined to improve this analysis and its interpretation, in particular a hierarchical classification of the macro-classes with a proposed typology of the classes that is easier to interpret. The Kohonen maps we obtained are less precise than classical MCA projections, but they summarize better the various relationships (attractions and repulsions) between the DPN-related variables and their modalities. Even if simultaneous representation of the various modalities and the individuals does not have a rigorous rationale, it nevertheless gives good results. These methods have the advantage of producing on the one hand a Kohonen map comparable with a traditional MCA projection and on the other hand a classification of the individuals due to the combination of the variables. Moreover, the method is particularly economical regarding computing time, which is a considerable advantage compared with classical methods such as hierarchical classification.

One of the main advantages of the proposed analysis is that the groups were not artificially classified according to DPN severity, because the available instruments do (e.g. the scores) are usually set and classes of severity created before analyzing the sample characteristics [13–18]. The proposed analysis in turn evaluates the composition of the sample characteristics and then groups them into macro-classes (groups) according to some chosen variables. We could identify the main groups that were formed and choose the most meaningful levels (separations or groups) according to the information being sought.

## Conclusions

Patients could be grouped in different macro-classes that represent different severities of the disease, including intermediate groups between healthy and severe conditions. The variables that most discriminated between groups were vibration perception rather than the traditional tactile sensitivity, pointing to a need for reconsideration of the current screening techniques. Calluses and cracks also do not discriminate severity status and should be interpreted together with other clinical DPN-related variables. The proposed methodology was shown to be a valuable tool to extract information regarding the specific contribution of each variable and to compare its discriminant power alongside other variables.
